# Robbery Victimization in Early Adulthood, and Depression and Anxiety at Age 30 Years: Results From the 1982 Pelotas (Brazil) Birth Cohort Study

**DOI:** 10.3389/fpubh.2022.821881

**Published:** 2022-06-09

**Authors:** Jesem Douglas Yamall Orellana, Joseph Murray, Natália Peixoto Lima, Ricardo Tavares Pinheiro, Bernardo Lessa Horta

**Affiliations:** ^1^Research Support Unit, Leônidas and Maria Deane Institut, Oswaldo Cruz Foundation, Manaus, Brazil; ^2^Postgraduate Program in Epidemiology, Federal University of Pelotas, Pelotas, Brazil; ^3^Human Development and Violence Research Centre, Federal University of Pelotas, Pelotas, Brazil; ^4^Postgraduate Program in Health and Behavior, Catholic University of Pelotas, Pelotas, Brazil

**Keywords:** violence, middle-income country, mental disorders, crime victims, cohort studies, Brazil, urban populations

## Abstract

Robbery is one of the most common urban crimes, but little is known about its relationship with mental disorders in young adults. This study aimed to assess the relationship between robbery victimization and Major Depressive Disorder (MDD), Generalized Anxiety Disorder (GAD) and comorbidity between MDD and GAD at 30 years of age. A birth cohort study has followed all children born in the city of Pelotas, southern Brazil, since 1982. At ages 23 and 30 years, participants were interviewed and asked about lifetime and recent experiences of robbery. Covariates were measured in interviews between birth and age 30 years. MDD and GAD were measured using the MINI-International Neuropsychiatric Interview. Adjusted prevalence ratio (aPR) and corresponding 95% confidence interval (CI) for associations between robbery and mental disorders were calculated using Poisson regression with robust standard error. Of 3,701 cohort members interviewed at age 30 years, 42% reported robbery victimization during their lifetime. Victimization across three periods (lifetime, past 10 years, past 12 months) was associated with increased occurrence of MDD, GAD, as well as the MDD and GAD comorbidity. The strongest associations were found to robbery occurring in the previous 12 months with the MDD and GAD comorbidity, both for burglary at home (aPR 2.52; 95% CI 1.52–4.22) or community family victimization (aPR 2.10; 95% CI 1.34–3.27). These findings highlight the importance of community violence for mental health in young adulthood, and the need for public policies to prevent violence as well as support services for victims to mitigate its adverse health consequences.

## Introduction

Mental disorders, mainly anxiety and depression are an important global public health problem (1); 10.5% of the burden of disease in Latin America and Caribbean is due to mental and behavioral disorders (2). In three Brazilian birth cohorts (Ribeirão Preto, São Luís and Pelotas), the prevalence of depression was higher than 7% and generalized anxiety was over 9% among adults aged from 22 to 39 years (3).

Several studies have shown that mental disorders in adulthood have roots in exposure to stressful events in childhood or adolescence, such as child maltreatment, neglect, domestic violence, physical abuse, bullying, familial members death and contexts affected by war or armed conflict (4–9). In adults, post-traumatic stress disorder related to experiences of robbery has been reported among convenience store and bank employees (10–12). Moreover, depression, anxiety and social phobia have been found to correlate with community violence (9, 13). High rates of violence in Latin America may be an important cause of mental disorder in the region (14). For example, a survey of adults in Rio de Janeiro and São Paulo reported that about one in every ten subjects had experienced traumatic experiences, especially those involving interpersonal violence in the last 12 months, which was strongly related to several mental disorders (15).

Robbery is one of the most important forms of exposure to community violence (16–20), and a previous study carried out in Pelotas found that robbery victimization was associated with depression and anxiety among adolescents (19), in line with other studies performed in high-income countries, such as United States and England (21, 22). There is, hence, a substantial set of findings from studies highlighting that community violence, mainly robbery victimization, may be related to mental disorders at different stages in the lifecourse, since robbery victims often report not only feelings of fear and concerns immediately afterwards the victimization, but also apprehension with possible revictimization and mental disorders occurrence, such as major depression and generalized anxiety, even months or years later, for example (13, 23–25).

Although several studies has already been evaluated the association between stressful life events or interpersonal/community violence and mental disorders (7, 13, 15), there is no studies that has evaluated the robbery exposure at different times in the life course (lifetime, in the last 10 years, in the last 12 months) with specific mental disorders, such as major depression, generalized anxiety and the MDD and GAD comorbidity at age 30. In its most general form, our hypothesis is that the robbery victimization is associated with mental disorders at different stages in the life course. Therefore, this study was aimed at assessing the relationship between robbery victimization and mental disorders at 30 years in a Brazilian town with high rate of violence.

## Materials and Methods

### Study Designs and Participants

In 1982, all maternity hospitals in Pelotas, a southern Brazilian city, were daily visited and births identified. Those livebirths whose families lived in the urban area of the city were examined (*n* = 5,914) and their mothers interviewed. These individuals have been prospectively followed at different ages. From June 2012 to February 2013, the cohort members were invited to attend the research clinic to be interviewed and examined (mean age: 30.2 years). Further details on the cohort methodology have been previously published (26, 27).

### Victimization Due to Interpersonal Violence

Participants were asked about experiences of robbery (“assalto”) any time in their lifetime, in the prior 10 years, and in the 12 months prior to interview at ages 23 and 30 years (Table 1). Four dichotomous variables were created about robbery up to age 30-years. Lifetime robbery up to age 30 years was coded positively if robbery was reported at either age 23 or 30. Robbery in the last 10 years up to age 30 was coded positively if that question was responded to positively at age 30, or if robbery in the previous 12 months was reported at age 23. Robbery in the last 12-months at age 30 years was less specific to the participant, and included two variables: first whether or not the participant or a family member had been “attacked or robbed” in the last 12 months (yes/no); second whether or not the household had been burgled in the past 12 months at age 30 years (yes/no).

**Table 1:** Description of the specific assessments covered by this study, according age, 1,982 Pelotas birth cohort, Brazil.

**Exposure**	**Assessment age**
Community familial victimization (over the last 12 months)	30 years
Burglary at home (over the last 12 months)	30 years
Robbery^a^ (over the past 10 years)	23 and 30 years
Robbery^a^ (lifetime)	23 and 30 years
**Outcome**
Mental disorders	30 years

^a^*See more details on Methods Section*.

### Assessment of Mental Disorders

Major Depressive Disorder (MDD) and Generalized Anxiety Disorder (GAD) were assessed by trained psychologists (Table 1) using the Mini International Neuropsychiatric Interview (MINI), a short semi-structured diagnostic interview for the fourth version of the Diagnostic and Statistical Manual of Mental Disorders (DSM-IV) and the International Classification of Diseases, 10th revision (ICD-10). Both MDD and GAD symptoms were determined by preestablished algorithms in MINI (28). The occurrence of MDD and GAD comorbidity was also considered in the analyses.

### Confounder Variables

The following variables were considered as possible confounders: sex (female; male); family income at birth (income tertiles); maternal age at birth (<20 years; 20–29 years; 30 years and over). Family income at birth, according to the minimum wage at the time, was originally collected in five categories in the perinatal study, and then transformed into a continuous variable through principal component analysis (PCA) using four variables (affiliation to the public health care insurance system at birth, maternal schooling, height and skin color) and finally the same continuous variable was converted in income tertiles (29). At 23 years, age at first alcohol use (<13 years; 13–22 years or never), race/skin color (self-reported) using the official Brazilian classification of ethnicity, and complete years of schooling (0–8; 9–11; ≥12) were evaluated. At 30 years, household density (one; two; three; four; five and more people), familial income in tertiles (Poorest; 2nd; Wealthier), and marital status (single; married/common-law marriage; separated/widowed) were evaluated. The familial income in tertiles at 30 years was exclusively used as possible confounder for association between victimization in the 12 months prior to interview and mental disorders just because this covariate could be temporally associated with victimization exposure at 30 years and not in the prior 10 years or lifetime.

### Statistical Analysis

Pearson's chi-square test was used to compare proportions. To estimate the prevalence ratio (PR) of mental disorders, Poisson regression, with a consistent covariance matrix estimator of the HC2 type was used (30). The final adjusted model considered as significant the association between exposure and outcome with significance set at 5%. Statistical analyses were performed in R program, version 3.3.2 (http://www.r-project.org).

## Results

In the 2012–13 visit, 3,701 individuals were interviewed at age 30 years. Considering also those who were identified as deceased, this represents a follow-up rate of 68.1%. Of those interviewed, 41.9% (1,528) reported at least one episode of robbery victimization in their lifetime, and 27.9% (1, 17) over the past 10 years. Regarding victimization in the last 12 months, 7.6% reported community victimization (of a family member) and 4.6% burglary at home. With regard to mental disorders at 30 years, 8.6% presented MDD, 9.9% GAD and 3.9% the MDD and GAD comorbidity (Table 2).

**Table 2:** Sociodemographic, perinatal, lifestyle, robbery victimization and mental disorders characteristics, 1,982 Pelotas birth cohort, Brazil.

	***n***	**%**
**Sociodemographic and perinatal characteristics**		
**Sex**		
Female	1,886	51.7
Male	1,763	48.3
**Familial income at birth (tertiles)**		
Poorest	1,144	31.3
2nd	1,298	35.6
Wealthier	1,207	33.1
**Maternal age at birth (years)**		
<20	547	15.0
20–29	2,094	57.4
≥30	1,007	27.6
**Race/skin color of the cohort member**		
White	2,506	75.0
Black	534	16.0
Brown	187	5.6
Others	116	3.4
**First time alcohol consumption at age 23 years (years)**		
<13	921	28.0
13–22 or never	2,366	72.0
**Years of schooling at age 23 years**		
0–8	1,155	15.6
9–11	1,668	49.9
≥12	520	15.6
**Familial income of minimum wage at age 30 years (tertiles)**		
Poorest	1,157	33.2
2nd	1,149	33.0
Wealthier	1,175	33.8
**Marital status at age 30 years**		
Single	1,090	29.9
Married/common-law marriage	2,410	66.1
Separated/widowed	147	4.0
**Household density at age 30 years (number of persons)**		
One	270	7.7
Two	939	25.8
Three	1,174	32.2
Four	721	19.8
Five or more	540	14.8
**Robbery victimization**		
Robbery (lifetime)	1,528	41.9
Robbery (in the last 10 years)	1,017	27.9
Community familial victimization (in the last 12 months)	279	7.6
Burglary at home (in the last 12 months)	169	4.6
**Mental disorders**		
Major Depressive Disorder (MDD)	314	8.6
Generalized Anxiety Disorder (GAD)	362	9.9
MDD and GAD comorbidity	144	3.9

Table 3 shows the relationships of indicators of victimization with sociodemographic, perinatal, lifestyle and mental disorders variables. In the bivariate analysis, the prevalence of lifetime robbery was higher among females, those in the highest tertile of family income at birth, with maternal age at birth between 20 and 29 years, which have reported white skin color, first time alcohol consumption before 13 years and schooling >11 years at age 23 years, as well as among single individuals, and in the highest tertile of family income at age 30 years (*p*-value < 0.05). The prevalence of robbery over the last 10 years was higher among female, those in the highest tertile of family income at birth, with schooling >11 years at age 23 years, as well as among singles individuals and lower household density at age 30 years (*p*-value < 0.05). MDD, GAD and the MDD and GAD comorbidity were not associated with lifetime robbery or over the past 10 years. However, the prevalence of MDD was higher among those who reported community family victimization over the past 12 months (*p*-value < 0.05) and burglary at home in the same period (*p*-value < 0.05), both for GAD and for MDD and GAD comorbidity the pattern of association was similar (Table 3).

**Table 3:** Frequency of robbery victimization, according to sociodemographic, perinatal, lifestyle and mental disorders at age 30 years, 1,982 Pelotas birth cohort, Brazil.

**Variables**	**Robbery (lifetime)**	**Robbery (over the past 10 years)**	**Burglary at home (over the last 12 months)**	**Community familial victimization (over the last 12 months)**
	***n* (%)**	***n* (%)**	***n* (%)**	***n* (%)**
**Sex**	*P* = 0.001	*P* = 0.001	*p* = 0.074	*p* = 0.273
Female	898 (50.9%)	544 (30.9%)	93 (5.3%)	126 (7.1%)
Male	630 (33.4%)	473 (25.1%)	76 (4.0%)	153 (8.1%)
**Familial income at birth**	*p* = 0.001	*p* = 0.024	*p* = 0.592	*p* = 0.446
Poorest	409 (35.8%)	299 (26.1%)	55 (4.8%)	96 (8.4%)
2nd	525 (40.4%)	347 (26.7%)	54 (4.2%)	91 (7.0%)
Wealthier	594 (49.2%)	371 (30.7%)	60 (5.0%)	92 (7.6%)
**Maternal age at birth (years)**	*p* = 0.036	*p* = 0.857	*p* = 0.243	*p* = 0.089
<20	217 (39.7%)	154 (28.2%)	32 (5.9%)	54 (9.9%)
20–29	915 (43.7%)	589 (28.1%)	97 (4.6%)	148 (7.1%)
≥30	396 (39.3%)	274 (27.2%)	40 (4.0%)	76 (7.5%)
**Race/skin color of the cohort member**	*p* = 0.001	*p* = 0.134	*p* = 0.913	*p* = 0.944
White	1,152 (46.0%)	724 (28.9%)	118 (4.7%)	190 (7.6%)
Black	190 (35.6%)	150 (28.1%)	25 (4.7%)	41 (7.7%)
Brown	61 (32.6%)	39 (20.9%)	8 (4.3%)	16 (8.6%)
Others	53 (45.7%)	32 (27.6%)	7 (6.0%)	10 (8.6%)
**First time alcohol consumption at age 23 years (years)**	*p* = 0.001	*p* = 0.050	*p* = 0.053	*p* = 0.871
<13	469 (50.9%)	285 (30.9%)	54 (5.9%)	72 (7.8%)
13–22 or never	973 (41.1%)	651 (27.5%)	101 (4.3%)	181 (7.7%)
**Years of schooling at age 23 years**	*p* = 0.001	*p* = 0.047	*p* = 0.704	*p* = 0.377
0–8	423 (36.6%)	296 (25.6%)	59 (5.1%)	96 (8.3%)
9–11	765 (45.9%)	497 (29.8%)	77 (4.6%)	128 (7.7%)
≥12	268 (51.5%)	152 (29.2%)	22 (4.2%)	33 (6.3%)
**Familial income of minimum wage at age 30 years (tertiles)**	*p* = 0.001	*p* = 0.259	*p* = 0.469	*p* = 0.546
Poorest	417 (36.0%)	304 (26.3%)	62 (5.4%)	88 (7.6%)
2nd	488 (42.5%)	325 (28.3%)	49 (4.3%)	96 (8.4%)
Wealthier	558 (47.5%)	344 (29.3%)	56 (4.8%)	84 (7.1%)
**Marital status at age 30 years**	*p* = 0.001	*p* = 0.001	*p* = 0.966	*p* = 0.778
Single	511 (46.9%)	378 (34.7%)	49 (4.5%)	79 (7.4%)
Married/common-law marriage	963 (40.0%)	605 (25.1%)	113 (4.7%)	189 (7.2%)
Separated/widowed	52 (35.4%)	33 (22.4%)	7 (4.8%)	10 (6.8%)
**Household density at age 30 years (number of persons)**	*p* = 0.001	*p* = 0.001	*p* = 0.849	*p* = 0.447
One	136 (50.4%)	100 (37.0%)	14 (5.2%)	18 (6.7%)
Two	441 (47.0%)	289 (30.8%)	42 (4.5%)	69 (7.3%)
Three	491 (41.8%)	330 (28.1%)	49 (4.2%)	81 (6.9%)
Four	269 (37.3%)	175 (24.3%)	37 (5.1%)	59 (8.2%)
Five or more	187 (12.3%)	121 (22.4%)	27 (5.0%)	50 (9.3%)
**Major Depressive Disorder (MDD)**	*p* = 0.512	*p* = 0.556	*p* = 0.007	*p* = 0.008
No	188 (8.9)	222 (8.4)	290 (8.3)	278 (8.2)
Yes	126 (8.2)	92 (9.3)	24 (14.2)	36 (12.9)
**Generalized Anxiety Disorder (GAD)**	*p* = 0.859	*p* = 0.261	*p* = 0.057	*p* = 0.001
No	212 (10.0)	252 (9.6)	338 (9.7)	319 (9.5)
Yes	150 (9.8)	110 (10.8)	24 (14.2)	43 (15.4)
**MDD and GAD**	*p* = 0.972	*p* = 0.523	*p* = 0.006	*p* = 0.007
No	83 (3.9)	100 (3.8)	130 (3.7)	124 (3.7)
Yes	61 (4.0)	44 (4.3)	14 (8.3)	20 (7.2)

*p-value to the chi-squared test*.

After controlling for confounders, all types of victimization experiences (lifetime, past 10 years, past 12 months, in the community and at home) were associated with MDD, GAD and their comorbidity (Table 4). The occurrence of MDD was 36% [adjusted PR (aPR); 95% CI 1.08–1.70] and 32% (aPR; 95% CI 1.04–1.68) higher among those who were robbery victims, ever or in the last 10 years, respectively. For victimization over the past 12 months, both for burglary at home or community family victimization, the occurrence of MDD was 88% (aPR; 95% CI 1.29–2.73) and 60% (aPR; 95% CI 1.15–2.21) higher, respectively (Table 4). The occurrence of GAD was 36% (aPR; 95% CI 1.10–1.67) and 39% (aPR; 95% CI 1.11–1.73) higher among those who were victims of lifetime robbery and robbery in the past 10 years, respectively. With respect to victimization over the past 12 months, both for burglary at home or community family victimization, the occurrence of GAD was 47% (aPR; 95% CI 1.01–2.15) and 66% (aPR; 95% CI 1.24–2.21) higher, respectively (Table 4). Finally, the occurrence of comorbidity of MDD and GAD was 57% (aPR; 95% CI 1.11–2.22) and 45% (aPR; 95% CI 1.01–2.10) higher among those with lifetime and over the past 10 years robbery victimization, respectively. For victimization over the past 12 months, both for burglary at home or community family victimization, the occurrence of MDD and GAD was 2.53 (aPR; 95% CI 1.52–4.22) and 2.10 (aPR; 95% CI 1.34–3.27) higher, respectively (Table 4).

**Table 4:** Relationship between robbery with Major Depressive Disorder (MDD), Generalized Anxiety Disorder (GAD) and MDD and GAD comorbidity, 1,982 Pelotas birth cohort, Brazil.

**Characteristics**	**MDD**		**GAD**		**MDD and GAD**	
	**Unadjusted**	**Adjusted**	**Unadjusted**	**Adjusted**	**Unadjusted**	**Adjusted**
	**PR^**a**^ (95% CI^**b**^)**	**PR^**a**^ (95% CI^**b**^)**	**PR^**a**^ (95% CI^**b**^)**	**PR^**a**^ (95% CI^**b**^)**	**PR (95% CI^**b**^)**	**PR^**a**^ (95% CI^**b**^)**
**Robbery (lifetime)**						
No	Ref^c^	Ref^c^	Ref^c^	Ref^c^	Ref^c^	Ref^c^
Yes	0.93 (0.75–1.15)	1.36 (1.08–1.70)^d^	0.98 (0.81–1.20)	1.36 (1.10–1.67)^d^	1.02 (0.74–1.41)	1.57 (1.11–2.22)^d^
**Robbery (over the past 10 years)**						
No	Ref^c^	Ref^c^	Ref^c^	Ref^c^	Ref^c^	Ref^c^
Yes	1.07 (0.85–1.35)	1.32 (1.04–1.68)^d^	1.13 (0.91–1.40)	1.39 (1.11–1.73)^d^	1.14 (0.80–1.61)	1.45 (1.01–2.10)^d^
**Burglary at home (over the last 12 months)**						
No	Ref^c^	Ref^c^	Ref^c^	Ref^c^	Ref^c^	Ref^c^
Yes	1.70 (1.16–2.51)	1.88 (1.29–2.73)^e^	1.46 (0.99–2.15)	1.47 (1.01–2.15)^e^	2.22 (1.30–3.77)	2.53 (1.52–4.22)^e^
**Community familial victimization (over the last 12 months)**						
No	Ref^c^	Ref^c^	Ref^c^	Ref^c^	Ref^c^	Ref^c^
Yes	1.56 (1.13–2.17)	1.60 (1.15–2.21)^e^	1.63 (1.21–2.19)	1.66 (1.24–2.21)^e^	1.95 (1.23–3.08)	2.10 (1.34–3.27)^e^

^a^*Prevalence ratio; ^b^Confidence interval; ^c^Reference group; ^d^Adjusted for sex, family income, Race/skin color of the cohort member, maternal age at birth, alcohol intake before 13 years old, years of schooling at age 23 years, marital status and household density at age 30 years; ^e^Adjusted for sex, family income, Race/skin color of the cohort member, maternal age at birth, alcohol intake before 13 years old, years of schooling at age 23 years, marital status, family income in tertiles and household density at age 30 years*.

## Discussion

To our knowledge, this is one of the few population-based studies to evaluate the association of robbery victimization with depression, anxiety and their comorbidity. And, 42% of the study participants had experienced robbery by age 30 years, and about two thirds of these individuals reported that robbery victimization had occurred between 20 and 30 years. Lifetime robbery victimization and over past 10 years, as well as community family victimization and burglary at home over the past 12 months was associated with increased occurrence of Major Depressive Disorder, Generalized Anxiety Disorder and their comorbidity.

The high prevalence of robbery victimization between 20 and 30 years may be related to exposure in environments that are favorable to robbery in young adulthood, including commercial streets, public squares or collective transport (31), and the rise of urban violence in Pelotas in recent years. But many age groups are exposed to robbery in the city. In the Pelotas 1993 Birth Cohort Study, it was found that robbery was one of the most highly registered violent crimes up to age 18 years (20), based on official records. Robbery is also one of the three most common crimes that result in imprisonment in Rio Grande do Sul, the province to which Pelotas city belongs (32). The prevalence of community family victimization and burglary at home over the past 12 months were also high in the current study, a relatively common pattern in low and middle-income countries (33–37).

It is noteworthy, that in 2030 nearly 60% of the world's population will be living in urban areas, responsible for more than 75% of the world's Gross Domestic Product (38, 39). Therefore, beyond its psychosocial impacts and the reduction in life expectancy associated with violence (1, 40–42), it seems equally important to consider its economic burden (43), especially in low and middle-income countries, where rates of violence are so high.

Crude (unadjusted) analyses did not reveal associations between mental disorders and robbery occurring over the past 10 years. However, after adjusting for confounders, significant associations were observed, consistent with the results of Murray et al. for 18 years old (19). The association with robbery victimization was higher for comorbidity of MDD and GAD, than that for MDD or GAD alone. For decades, research has identified the effects of violence in the home on mental disorders (13, 44), but less attention has been given to the specific effects of events such as robbery in the community (19). This is an important issue, especially in contexts where community violence is common.

The prevalence of MDD, GAD, as well as MDD and GAD comorbidity was higher among those who reported at least one episode of community family violence in the last 12 months, as well as among the victims of burglary at home. This is consistent with other Brazilian studies. A cohort study with workers from a Brazilian university showed a higher occurrence of common mental disorder among those who reported having been victims of robbery (aOR = 1.5; 95% CI 1.2–1.8) (45) and higher psychological stress for those who reported direct violence victimization in the last 12 months (aOR 1.6; 95% CI 1.0–2.4) (40). A study with individuals aged 15–24 years in São Paulo city observed an association of violent victimization in the last 12 months with anxiety symptoms (aOR 1.68; 95% CI 1.01–2.78) and MDD (aOR 2.27; 95% CI 1.09–4.74) (36). Similarly, a study carried out in 2007–2008 in Rio de Janeiro and São Paulo cities (15), observed an association of exposure to traumatic events over the past 12 months with MDD (aOR 1.20; 95% CI 1.15–1.24), GAD (aOR 1.19; 95% CI 1.12–1.26) and post-traumatic stress disorder (TSPT) (aOR 1.30; 95% CI 1.24–1.36).

Our results are also consistent with studies in other locations. A study carried out with adults in United States (21) reported an association of lifetime robbery with anxiety (aOR 1.38; 95% CI 1.04–1.76) and mood disorders (aOR 1.31; 95% CI 1.07–1.59). In addition, a study carried out in London (22) showed that adults exposed to lifetime violent victimization (attacks, burglary, robbery, physical aggression and sexual violence) had greater odds of common mental disorder (aOR 2.86; 95% CI 1.82–4.49) and post-traumatic stress disorder (aOR 13.38; 95% CI 5.13–34.90), reinforcing that community violence may be related to mental disorders.

Victims report that robbery is a very frightening experience, contributing to fear of future community violence (23), and robbery victimization is usually related to intensification of anguish feelings, worry and fear (23). Our results indicate that the magnitude of association with robbery was slightly greater for GAD, in comparison to MDD, regardless of the time period evaluated. As fear and excessive worry are symptoms frequently associated with anxiety (24), the stronger association observed for robbery and GAD could be partially explained by feelings of fear and related concerns of further robbery victimization (25).

Several mechanisms have been proposed to explain the relationship between community violence and mental disorders throughout the lifecourse (46, 47). The short and long-term effects of robbery on mental disorders have been mostly discussed based on different pathophysiological responses focused on trauma (19) or stressful events with the potential to modify the functioning of the sympathetic nervous system and the axis hypothalamic-pituitary-adrenal (HPA) (24, 48), inflammatory mechanisms in the brain and peripherally (49) or even with epigenetic mechanisms (50). Regarding depression and anxiety disorders, HPA axis hyperactivity has been the main pathophysiological mechanism associated with stressful events resulting from interpersonal violence, at different stages in the lifecourse (51–53). In addition, there are also the approach to psychological mechanisms, where its impairment functioning due to early trauma would leading to limited response capacity in the face up to stress experiences lifelong (7, 47, 54).

Lifetime robbery and over the past 10 years was more common among females, as well as in white, single individuals, living in households with lower household density and among individuals whose family income at birth was in the highest income tertile. These results are in line with other literature on non-lethal community violence victimization (19, 21, 55, 56). Unlike the characteristics associated with non-lethal community violence victimization, homicide is more frequent among poor males and those of black race/skin color (57–60). This opposite pattern observed for robbery victims is, in some sense, expected because is presumed that the offender overall seeks to select apparently richest people, either on his physical appearance, clothing items, type of goods or places frequented.

This study has some limitations, the term “assalto” is commonly used in Brazil and it can be translated as “robbery.” But it does not necessarily correspond to an episode of robbery, as it is also associated with theft, a type of crime in which there is no threatening contact between the victim and the aggressor. Therefore, theft victimization might have underestimated the magnitude of the observed associations, its potential for psychological trauma is lower than robbery, because the victim is prone not only to threat or intimidation, but also to serious physical aggression, for example. Because we did not collect data on the use of weapon during the victimization, nor on the context or number of episodes of robbery victimization, we were not able to evaluate the impact of exposure severity. Therefore, even though we have evaluated the exposures at different times (lifetime, in the last 10 years, and the last 12 months) and its association with mental disorders at 30 years of age, probably softening the temporality problem, we have no way of knowing whether the investigated mental disorders outcomes were already present at the time of exposure. Finally, although we have handled data the Pelotas 1982 birth cohort study, our analysis was focused in data at birth, 23 and 30 years old, because on previous follow-up visits, such as those which have been performed between 6 and 19 years old, has not been possible gathering more detailed information about early life stress events (e.g., childhood and adolescence) to the whole sample in any of these follow-up visits. Therefore, it is possible that the lack of on the domestic environment during childhood and adolescence, especially child maltreatment, as sexual, physical or emotional abuse, or other types of early life stress events as death of a family member, which are potential confounders, may have overestimated the magnitude of the associations. Strengths of this study include the high follow-up rate at age 30 years (68.1%), the large sample size, the assessment of mental disorders using diagnostic instruments, reducing the likelihood of classification error.

The results of this study reinforce prior findings on the influence of recent episodes of community violence on the occurrence of mental disorders, but also points to a possible influence of long-term effects on depression and anxiety in young adults. Given the importance of community violence for mental disorders in increasingly urbanized cities, it is desirable that stakeholders also increase its efforts to preventing, reducing or mitigating the consequences of exposure to community violence, offering support services for victims, mainly in low and middle-income countries where socioeconomic and access to health services determinants play an important role.

## Data Availability Statement

The raw data supporting the conclusions of this article will be made available by the authors, without undue reservation.

## Ethics Statement

The studies involving human participants were reviewed and approved by Research Ethics Committee of the School of Medicine, Federal University of Pelotas. Written informed consent to participate in this study was provided by the participants' legal guardian/next of kin.

## Author Contributions

JO, JM, and BH contributed to conceptualization, analysis, and organized the findings and subsequently put the information in a manuscript format. NL organized the database and was involved in the data analysis, figure tables, and the writing of the manuscript. RP contributed to organization, data analysis, and the writing of the manuscript. All authors contributed to the article and approved the submitted version.

## Funding

The research project was partially financed by Wellcome, Award Number 086974/Z/08/Z and the Joe's current Wellcome Investigator Award Number is 210735/Z/18/Z. This research was funded in part, by the Graduate Studies Coordinating Board (Capes), funding code 001, and is currently financed by the Science of Technology Department, Brazilian Ministry of Health.

## Conflict of Interest

The authors declare that the research was conducted in the absence of any commercial or financial relationships that could be construed as a potential conflict of interest.

## Publisher's Note

All claims expressed in this article are solely those of the authors and do not necessarily represent those of their affiliated organizations, or those of the publisher, the editors and the reviewers. Any product that may be evaluated in this article, or claim that may be made by its manufacturer, is not guaranteed or endorsed by the publisher.
